# Diagnostic Value of Interferon-Gamma Release Assays for Tuberculosis in the Immunocompromised Population

**DOI:** 10.3390/diagnostics12020453

**Published:** 2022-02-10

**Authors:** Ying Yang, Hong-Jiao Wang, Wei-Lin Hu, Guan-Nan Bai, Chun-Zhen Hua

**Affiliations:** 1 Department of Infectious Diseases, The Children’s Hospital, Zhejiang University School of Medicine, National Clinical Research Center for Child Health, Hangzhou 310052, China; 6510124@zju.edu.cn (Y.Y.); 6513333@zju.edu.cn (H.-J.W.); huweilin@zju.edu.cn (W.-L.H.); guannanbai@zju.edu.cn (G.-N.B.); 2 Department of Medical Microbiology, The Children’s Hospital, Zhejiang University School of Medicine, National Clinical Research Center for Child Health, Hangzhou 310052, China

**Keywords:** interferon-gamma release assays, *Mycobacterium tuberculosis*, the immunocompromised population, diagnostic value

## Abstract

Interferon-gamma release assays (IGRAs) are widely used in the diagnosis of *Mycobacterium tuberculosis* (*M. tuberculosis*) infection by detecting interferon-γ released by previously sensitized T-cells in-vitro. Currently, there are two assays based on either enzyme-linked immunosorbent assay (ELISA) or enzyme-linked immunospot (ELISPOT) technology, with several generations of products available. The diagnostic value of IGRAs in the immunocompromised population is significantly different from that in the immunocompetent population because their results are strongly affected by the host immune function. Both physiological and pathological factors can lead to an immunocompromised situation. We summarized the diagnostic value and clinical recommendations of IGRAs for different immunocompromised populations, including peoplewith physiological factors (pregnant and puerperal women, children, and older people), as well as people with pathological factors (solid organ transplantation recipients, combination with human immunodeficiency virus infection, diabetes mellitus, end-stage renal disease, end-stage liver disease, and chronic immune-mediated inflammatory diseases). Though the performance of IGRAs is not perfect and often requires a combination with other diagnostic strategies, it still has some value in the immunocompromised population. Hopefully, the newly developed IGRAs could better target this population.

## 1. Introduction

Interferon-gamma release assays (IGRAs) are widely-used technology in diagnosing *Mycobacterium tuberculosis* (*M. tuberculosis*) infection. At present, a total of 25 countries (including both developed and developing countries) have issued guidelines on IGRAs for the diagnosis of latent tuberculosis infection (LTBI) or the auxiliary diagnosis of active tuberculosis infection (ATBI) in children. *M. tuberculosis* can lead to the long-term presence of immune-specific central-memory T-cells (CCR7+CD27+) and effector-memory T-cells (CCR7−CD27−) in the human body. These two types of T-cells can be rapidly activated and proliferated upon the re-exposure to antigen and release interferon-γ (IFN-γ), which can be recognized by in vitro immunodiagnostic tests. The two specific antigens of *M. tuberculosis* are the 6 kDM early-secreted antigenic target protein (ESAT-6) and the 10 kD culture filtrate protein (CFP10), encoded in the region of difference 1, which is not present in Bacillus Calmette-Guérin (BCG) or most non-tuberculosis mycobacteria (NTMs). IGRAs facilitate the diagnosis of tuberculosis infection by detecting IFN-γ released from T-cells that are stimulated by two or more antigens. At present, there are two types of IGRAs, which are based on the enzyme-linked immunosorbent assay (ELISA) and enzyme-linked immunospot (ELISPOT) technology, respectively. The ELISA-based technology measures the amount of IFN-γ released by T-cells, while the ELISPOT-based technology measures T-cells that produce IFN-γ. They both have multiple generations of products available. For example, the T cell spot (T-SPOT) and Heparin-Binding Hemagglutinin-Induced TSPOT (HBHA-TSPOT) are ELISPOT-based products; the QuantiFERON-TB Gold (QFT-G), QuantiFERON-TB Gold In-Tube (QFT-GIT), and QuantiFERON-TB Gold Plus (QFT-Plus) are ELISA-based products.

The diagnostic value of IGRAs in the immunocompetent population was well-established. Compared with another cellular-immunity-based test (tuberculin skin test (TST)), it has comparable or higher sensitivity but significantly higher specificity. IGRAs can replace the TST in some specific populations, such as patients ready to receive tumor necrosis factor-α (TNF-α) blockers in the guidelines of a few countries. However, in the immunocompromised population, situations are complex and various. They are prone to having more *M. tuberculosis* reactivation, atypical clinical presentation, rapid progression, and worse prognosis. When an immune function, especially cellular immunity, is impaired, both the sensitivity and specificity of IGRAs are affected to varying degrees and the number of indeterminate results increases. The early diagnosis and treatment of *M. tuberculosis* infection in this population becomes more urgent and difficult. The diagnostic value of IGRAs varies due to different kinds of immunocompromised factors. We discuss this in the following sections for both physiologically and pathologically immunocompromised populations.

## 2. Discussion

### 2.1. People with Physiologically Immunocompromised Factors

#### 2.1.1. Pregnant and Puerperal Women

ATBI during pregnancy remains associated with poor maternal and fetal outcomes globally, including a 3 times increase in maternal morbidity, 9 times increase in miscarriage, 2 times increase in preterm birth and low birth weight, and 6 times increase in perinatal death [1]. Meanwhile, severe immune reconstitution inflammatory syndrome may occur in the postnatal period [2]. However, the diagnosis of ATBI during pregnancy can be difficult because of the atypical clinical presentation [3]. At the same time, LTBI is easy to be underestimated and its incidence varies by race and economic status. According to epidemiological findings, LTBI occurred in 14–48% of pregnant women in the United States [4]. LTBI during pregnancy and the puerperium may develop into ATBI due to a physiological suppression of the T-helper 1 (Th1) pro-inflammatory response [5]. A high incidence of ATBI was reported within 180 days postpartum, and the postpartum diagnosis reflects the onset of active prenatal disease [4]. Therefore, it is important to monitor *M. tuberculosis* infection during pregnancy. However, the suppression of Th1 cells significantly affects the performance of IGRAs and the TST.

The efficacy of IGRAs compared with the TST for diagnosing LTBI during pregnancy varied in different studies. In both low and high prevalence areas, IGRAs are more specific than the TST due to there being no cross-reaction with BCG [4]. In terms of sensitivity, most studies showed little difference between the two, but IGRAs were less sensitive under certain conditions. The reason may be that as time from *M. tuberculosis* exposure increases, the sensitivity of IGRAs decreases [6]. A meta-analysis that involved 22 studies showed 77–91% concordance between IGRAs and the TST [4]. Recent evidence suggested that IGRAs were a good predictive tool for ATBI and that a positive IGRA result or a recently elevated IGRA strongly predicted the risk of reactivation, even among strongly immunosuppressed women [7,8]. A study showed that IGRAs also had a higher sensitivity (84.5–100.0%) and specificity (37.6–96.4%) than the TST for ATBI [9], though they have not been approved for the diagnosis of ATBI in this population

However, the use of IGRAs has some issues. First, a higher proportion of indeterminate results during pregnancy may be due to a low mitogen response, which was reported to be around 4–6% [10]. Fortunately, this is often temporary, and repeated testing after 1–2 months is essential [11]. Second, the cut-off value for this population is not clear, which needs further exploring [12]. Third, it was reported that IFN-γ released during pregnancy and the postpartum period varied and the conversion rate of IGRAs was relatively high [13,14]. Therefore, the optimal time for IGRAs remains an unresolved issue. Despite its lower efficacy compared with non-pregnant populations, it is still the first recommendation in both high and low prevalence areas and is highly cost-effective. The TST is even more influenced than IGRAs due to skin anergy [14,15]. In addition, IGRAs are an important tool for screening LTBI in low-income areas where follow-up rates are extremely low. Some women have only one medical visit for their whole life (IGRAs require one visit only, while the TST requires two visits). A new generation of IGRAs (QFT-Plus) promises to improve the detection of LTBI during pregnancy, which detects IFN-γ secretion from both CD4+ and CD8 + T-cells [12]. This is based on the reduction in CD4 + T-cells without altering CD8 + T-cells in pregnant women [16].

#### 2.1.2. Older People

The elderly are an easily overlooked group that is vulnerable to *M. tuberculosis* infection. Epidemiological surveys over the past century showed they had the highest proportion of LTBI, possibly related to exposure to *M. tuberculosis* early in their lives [17]. The risk of progression to ATBI is 1.5 times higher than adults [18] due to a dysfunctional immune system marked by inflammation and oxidative stress [19]. ATBI in the elderly is often the result of reactivation rather than primary infection. They are prone to have poor treatment response and high mortality (up to 20–30%), which is 6 times higher than that of adults [20].

Due to the lack of a gold standard for the diagnosis of LTBI, it is hard to evaluate the sensitivity and specificity of IGRAs and the TST. No significant decrease in the positive rate of IGRAs was found in the elderly, while the positive rate of the TST gradually decreased due to the decline in skin immunity [18]. IGRAs from older people had a poorer concordance compared with adults [18]. Higher indeterminate rates (5–40%) in older people were found when compared with younger adults. The high proportion of indeterminate results is related not only to a lower mitogen response but also to a higher IFN-γ background (high baseline inflammatory status) [18]. Despite indeterminate or borderline results on initial IGRAs, a second attempt with another IGRA seems to resolve the problem [18]. LTBI in the elderly is highly heterogeneous and may arise from recent or very distant *M. tuberculosis* infection. Unfortunately, neither the TST nor IGRAs can distinguish between recent and remote infections [21,22].

ATBI in older people is often clinically and radiographically atypical because of multi-underline diseases. Furthermore, difficulties in sputum production make it difficult to obtain microbiological evidence. Therefore, immunological tests become extremely important [20]. In the diagnosis of ATBI, we found a significant decrease in the sensitivity of the TST and IGRAs [23]. The decline in the TST is more significant. A Japanese study on people over 80 years old showed that the sensitivity of IGRAs (67%) was higher than that of the TST (27%). High proportions of the indeterminate results (up to 33.3%) were also found in ATBI [23]. It is impractical to use an IGRA alone to diagnose ATBI in older people and a combination of tests is required. QFT-Plus was reported to perform well in clinical trials in the elderly, with significantly improved sensitivity [24].

#### 2.1.3. Children

Every year, over 7.6 million children under 15 years old worldwide are infected with *M. tuberculosis* and 1 million of them develop ATBI, with nearly 140,000 deaths [25]. Young children are at high risk of developing fatal, disseminated tuberculosis, accounting for nearly 20% of all tuberculosis-related deaths in high-burden countries [26]. Infants, young children, and adolescents have a greater risk of developing ATBI rather than primary school children. The risk of progression in infants is 40–50%, with 15% developing meningitis or disseminated disease; the risk of children aged 1–2 years is reduced to 25%, with 5% developing meningitis or disseminated disease [27,28]. Due to their difficulty in expectoration and concerns about invasive operation, the diagnosis of children often lacks a microbiological basis. This means that they lack the absolute evidence to evaluate the accuracy of the TST and IGRAs.

Among children more than 5 years old, the diagnostic value of IGRAs is positive. Though IGRAs do not have enough sensitivity and specificity to diagnose or exclude ATBI [29], it is an important auxiliary diagnostic technology. The sensitivity of IGRAs has no obvious advantage over the TST, but the specificity of IGRAs is higher [30,31]. Sensitivities were 78–93% for ELISA, 58–93% for ELISPOT, and 82–100% for the TST, while the specificities for IGRAs were 98–100% [32]. In unvaccinated children, concordance between TSTs and IGRAs was high (*κ* = 0.802), whereas discordance was mostly seen in the BCG-vaccinated children [33]. With anti-tuberculosis therapy, IGRAs were seen to switch from positive to negative in most patients, but it is not possible to rely on an IGRA monitoring treatment response for individuals [34,35,36]. In terms of LTBI, the concordance between the two is fairly poor, and the results of TST+/IGRA− often appear [37]. When risk factors are present, children with TST+/IGRA− may develop into ATBI. When children lack risk factors, those with TST+/IGRA− may be falsely positive due to BCG. In countries with high BCG coverage and high rates of NTMs infection, IGRAs are more reliable than the TST [38].

Using IGRAs for the diagnosis of *M. tuberculosis* infection in children under 5 is controversial. First, there is concern about the reduced sensitivity of IGRAs. A study from the United States showed that the sensitivity of IGRAs was low in the diagnosis of laboratory-confirmed ATBI among children under 5 years of age, with 58.7% positivity in the 2–4-years-old group and 51.9% positivity in the under-2-years-old group [39]. Despite this, there is a high level of concordance between IGRAs and the TST in the diagnosis of ATBI [33]. Different studies had different conclusions regarding the value of IGRAs and the TST for diagnosing LTBI. A cohort study of BCG-vaccinated children younger than 5 years showed that there was a high discordance (12.3%) between IGRAs and the TST, mostly TST+/IGRA− [40]; another study showed that for high-risk children aged 2–5 years old, IGRAs’ positivity was lower than that of the TST [41]. None of the TST+/IGRA− children developed into ATBI during the follow-up even without prophylactic treatment, demonstrating the false positivity of the TST that came from the cross-reaction with BCG [40,41,42]. Using the TST as a standard, a meta-analysis showed that IGRAs had higher sensitivity and specificity, with the pooled sensitivity and specificity of ELISA being 84.1 and 89.5%, respectively, and the pooled sensitivity and specificity of ELISPOT were 93.1 and 76.7%, respectively [43]. An IFN-γ-inducible protein 10 (IP-10) assay compared with current IGRAs will improve the diagnosis of LTBI in patients younger than 5 years [44]. Another reason to hesitate to use IGRAs is that younger age will significantly increase indeterminate results due to the low mitogen response caused by immunologic immaturity [45,46]. Indeterminate results in children under 2 years old can reach up to 40%. In addition, iron deficiency anemia, helminths, and malaria infection, which are common in children under 5 years old, may also increase the indeterminate rates [45]. However, the largest study found less than 1% of results were indeterminate, where the authors included more than 5000 IGRA results in children under 2 years old [47].

At present, an increasing number of guidelines approve the application of IGRAs in the diagnosis of LTBI in children. When high specificity is required (e.g., BCG-vaccinated children in low-risk areas), IGRAs are a better choice. When sensitivity is the first consideration (e.g., children about to receive immunomodulatory biologics), a TST and IGRA need to be performed simultaneously, and any positive result should be regarded as infection. However, for the diagnosis of ATBI in children, IGRAs are thought to be a complementary diagnostic technology [48,49].

### 2.2. People with Pathologically Immunocompromised Factors

#### 2.2.1. Human Immunodeficiency Virus Infection

The co-infection of human immunodeficiency virus (HIV) and *M. tuberculosis* be-comes a deathly combination. HIV increases the risk of tuberculosis disease progression, while *M. tuberculosis* reduces CD4 + T-cells recovery and increases acquired immune deficiency syndrome progression. Globally, tuberculosis is the leading cause of death for persons living with HIV, accounting for a third (208,000) of the estimated 690,000 HIV-related deaths in 2019 [50]. For the people co-infected with *M. tuberculosis* and HIV, the risk of progression to ATBI is 10% annually [51]. Data showed that HIV-infected people had up to 23 times more tuberculosis progression than non-HIV-infected ones and a higher risk of developing multidrug-resistant tuberculosis [52] and extrapulmonary and disseminated diseases [50]. Even when the level of CD4 + T-cells is high, there is still an increased risk. This suggests that the increased risk is not only because of CD4 + T-cells depletion but also the functional impairment of *M. tuberculosis*-specific T-cells. It also indicates that HIV damages the innate immune system [53]. Therefore, the screening of LTBI and the early diagnosis of ATBI are important for this population.

The clinical manifestation of ATBI in patients with HIV is variable and depends on the degree of immunosuppression. Cough, fever, night sweats, and weight loss are common and nonspecific. Fewer cavities are found in pulmonary tuberculosis, while extrapulmonary tuberculosis commonly involves the pleura, lymph node, central nervous system, abdominal organs, pericardium, and bone [50]. IGRAs have suboptimal sensitivity and specificity in the diagnosis of ATBI in HIV-infected people. A meta-analysis showed that the pooled sensitivities of GFT-GIT and T-SPOT were 76.7 and 77.4%, respectively, while the specificities were 76.1 and 63.1%, respectively [54]. Another meta-analysis showed that the pooled sensitivities of GFT-GIT and T-SPOT were 61 and 65%, respectively, while the specificities were 72 and 70%, respectively [55]. The sensitivity and specificity of low/middle-income countries are slightly lower than those of high-income countries [54]. At a low level of CD4 + T-cells, the ELISPOT method is more sensitive than that of the ELISA method [56]. IGRAs are similar to the TST regarding sensitivity [57,58]. The pooled indeterminate rates were 8.2% for ELISA and 5.9% for ELISPOT. In some studies, indeterminate results were reported to be up to 16% [54]. They are higher in high-burden settings and low-income counties than in low-burden settings and high-income countries [55,59]. The proportion of indeterminate results increases in patients with low CD4 + T-cells, especially when it is less than 200 cells/μL [55,60,61]. The ability of IGRAs for diagnosing ATBI is limited because of the high proportion of indeterminate results, which may result in over 10% of the patients being missed [62]. The combination of HBHA-TSPOT may help to evaluate the anti-tuberculosis response [63].

As for the diagnosis of LTBI, both IGRAs and the TST are affected by immunosuppression situations. However, some IGRAs (ELISPOT) are less affected than the TST [57,64]. Spanish guidelines recommend both IGRAs and the TST for LTBI screening among patients with HIV, but only IGRAs are recommended when CD4 + T-cells are below 200 cells/μL [49]. The sensitivities of ELISA and the TST decrease with the decrease in CD4 + T-cells, but not ELISPOT [64,65]. A meta-analysis found that the sensitivity of ELISPOT (72%, 95 CI 62–81%) was higher than that of ELISA (61%, 95 CI 41–75%) in this population [57]. Overall, the concordance between IGRAs and the TST is medium or poor [66,67]. Concordance is worse in low-income countries and patients with a low level of CD4 + T-cells [68]. IGRA+/TST− is a more common discordant result, suggesting that IGRAs are more sensitive than the TST [57,65,69]. Neither the TST nor IGRAs can predict the risk of ATBI well [70,71]. There are different conclusions about the predictive ability of IGRAs due to different follow-up periods, but at least patients with negative IGRAs have a low risk of ATBI in a short time [55,72]. Using it for predicting ATBI was reported to be cost-effective in some countries [73]. In addition, IGRAs are more convenient (one visit only) and improve screening rates for LTBI [74]. Affected by the level of CD4 + T-cells [60], the rate of indeterminate IGRAs results is quite high (up to 14%) [75], which are more common in ELISA than ELISPOT. In summary, all HIV-infected populations should be screened for LTBI and screened annually when CD4 + T-cells are below 200 cells/μL. The TST and IGRAs are both suboptimal, and the combination of the two may improve the diagnostic sensitivity [76]. Each country needs to judge which strategy is more appropriate in its national conditions.

#### 2.2.2. Solid Organ Transplant Recipients

For solid organ transplantation (SOT) recipients, the proportion of ATBI is very high due to their lifelong immunosuppression, which is about 4–30 times that of the general population. In most developed countries, the incidence of ATBI after SOT is 1.2–2.4%, and it can reach 12% in highly endemic regions [77]. It also significantly increases mortality (up to 30%) compared with general ATBI (<5%) [78]. ATBI of SOT recipients could come from the reactivation of LTBI from themselves and organ donors [78]. The clinical feature is a high proportion of extrapulmonary and disseminated tuberculosis, often involving transplanted organs. Symptoms are nonspecific, with fever as the common manifestation and imaging characterized by lobar infiltrates and few cavities. The risk of ATBI varies due to various grafts, with lung transplant recipients being at the highest risk.

Most international guidelines recommend screening for LTBI in all kinds of SOT candidates/donors, and both the TST and IGRAs are recommended as screening tools. Different studies showed great differences in the diagnostic value of the TST and IGRAs, which may be due to different immunosuppressive states caused by various organ failures [79]. A study showed that the sensitivity of the TST was surprisingly low in this population, with only 20–25% of post-transplant ATBI cases having a positive response to the TST before SOT [80]. Even with the two-step TST (boosted TST), the detection rate increased by only 4% [81]. IGRAs have been used to replace the TST in this scenario. Though the sensitivity of IGRAs is also affected, it has higher specificity in the BCG-vaccinated population. In addition, studies showed that IGRAs were highly sensitive among kidney and liver transplantation candidates [81,82]. A study of kidney transplant recipients showed that IGRAs could predict the risk of post-transplant ATBI in patients with a negative TST before transplantation [82]. Therefore, IGRAs are suitable for screening for LTBI before transplantation, particularly in BCG-vaccinated populations and liver and kidney transplant candidates. A study from Japan showed that screening SOT candidates using IGRAs was the most cost-effective method compared with the TST or no screening [83]. Interestingly, the intermediate IGRA results may vary by graft type: 41% of patients before liver transplantation had intermediate results compared with only 12% of patients before non-liver transplantation. Among IGRAs, ELISPOT seems to have a higher sensitivity than ELISA [84]. Due to the performance of IGRAs, a routine chest X-ray or computed tomography is recommended to find more evidence.

ATBI in SOT recipients is always atypical in clinical manifestation and has a high frequency of co-infections (cytomegalovirus infection, community-acquired pneumonia, urinary tract infections, Nocardia and Aspergillosis), which may lead to a delayed diagnosis [80]. A nucleic acid amplification test, mycobacterial culture, and histopathology are necessary. In contrast, the TST and IGRAs alone are unreliable, as they have reduced sensitivity in this population and do not differentiate between latent and active disease [85].

#### 2.2.3. Chronic Immuno-Mediated Inflammatory Diseases

Patients with chronic immune-mediated inflammatory diseases (IMIDs), including rheumatoid arthritis (RA), spongyl arthropathies (SpA), Crohn’s disease (CD), psoriasis, and juvenile idiopathic arthritis (JIA), have an increased primary tuberculosis infection and reactivation of LTBI. The immunosuppressive therapy (TNF-α blockers, glucocorticoids, etc.) in these patients exacerbates the situation [86].

Screening for LTBI is recommended before immunosuppressive therapy [49]. Although most of the available information is from small studies, it does, nevertheless, provide some indication of the performance of IGRAs in this population. Most studies found that the concordance between IGRAs and the TST was poor [87,88,89]. In some countries with low BCG coverage and low tuberculosis prevalence, the concordance will be higher [90]. Positive IGRA results were more common than for the TST, and even up to 50% of the patients were missed by the TST [87]. This may be because IGRAs detect IFN-γ in vitro only, while the TST is an in vivo test that needs a variety of cytokines, including IFN-γ. All these cytokines are easily inhibited by immune-suppressive medication due to the high false-negative rate of the TST. TST+/IGRA− was often found in the BCG-vaccinated population [89,91]. In addition, positive IGRAs were found to be associated with the risk factors of LTBI [89], but the positive predictive value still needs to be evaluated. It was reported that the proportion of indeterminate results in these populations was about 0–10.3% [91]. Therefore, IGRAs are superior to the TST in the diagnosis of LTBI before immunosuppressive treatment. It was reported that a TST followed by IGRAs was the best screening strategy if the TST was positive [92]. Of course, the conclusions may be different in areas with different tuberculosis burdens and BCG converges. Continuous IGRAs may provide greater diagnostic value [86].

The TST and IGRA are acceptable for LTBI screening in individuals with IMIDs. For example, the World Health Organization (WHO) guidelines recommend either of the two but do not explicitly address the choice of LTBI screening test [93]. However, there is a strong trend where more and more countries tend to use IGRAs rather than the TST. The Danish Society for Gastroenterology recommends IGRAs as the best indirect test of LTBI in individuals that are about to receive TNF-α blockers [94]. In the countries such as the United States, Canada, Australia, Japan, and some Western European countries (Britain, Italy, Germany, and Switzerland), IGRAs are served as routine tests [95]. Other countries suggest that when the risk of tuberculosis infection is high, IGRAs and the TST can be performed simultaneously to improve the sensitivity in this population, especially before TNF-α blocker treatment [48,49]. The exposure history and chest imaging are central to interpreting the TST/IGRA result and in determining the overall risk of LTBI in IMIDs [48]. ATBI cannot be diagnosed or ruled out by IGRAs or the TST in this population [48,49,93,96].

However, it is difficult to evaluate whether IGRAs are more effective than the TST in patients that are already receiving immunosuppressive therapy, as results from different studies varied [89]. Immunosuppressive drugs can significantly affect the performance of IGRAs, including sensitivity and indeterminate results. Glucocorticoids, for example, are positively associated with indeterminate results when the steroid dose is more than 7.5 mg prednisone equivalent. We believe this may be due to corticosteroid-induced lymphopenia or the impaired function of T-cells or antigen-presenting cells [97]. The positive rate of IGRAs was significantly lower in patients treated with TNF-α blockers [89].

#### 2.2.4. End-Stage Renal Disease

It is well-known that end-stage renal disease (ESRD) can lead to immune depression, such as the abnormal functioning of monocytes, neutrophils, and dendritic cells, which are directly associated with the risk of infection [98]. Among these patients, 63% of them undergo hemodialysis, which further exacerbates the immune function. Both cellular and humoral immune systems are affected, with the incidence of ATBI being 8–25 times higher and mortality being 2–3 times higher than in the general population from different studies [99,100].

The prevalence of LTBI in patients with ESRD varies (20–70%). The range was fairly wide because of the different tuberculosis burden settings and different detection methods. Most studies suggested that IGRAs were preferable for the TST. First, IGRAs have higher sensitivity and specificity. Compared with the TST, IGRAs using ELISA had a pooled sensitivity of 53 versus 31% and a pooled specificity of 69 versus 63%. IGRAs using ELISPOT also had higher sensitivity (50 vs. 31%) and specificity (67 vs. 63%) than the TST [101]. This relatively high sensitivity may be due to the detection of IFN-γ in vitro being less affected by uremic immunosuppression than in vivo [99]. It is noteworthy that peritoneal dialysis has a great effect on the performance of IGRAs, but hemodialysis does not [102]. Second, it is interesting to note that a positive IGRA result is more likely to be associated with clinical risk factors for LTBI, such as recent contact with a tuberculosis patient [103]. Nevertheless, IGRAs are still considered imperfect in ESRD because of their low diagnostic power for remote LTBI. HBHA-TSPOT seems to be more suitable for this population and can detect both recent and remote infections perfectly [104].

The diagnosis of ATBI in patients with ESRD is quite challenging because they always present atypical clinical and imaging manifestations, such as a high proportion of extrapulmonary tuberculosis, tuberculous pleurisy, tuberculous peritonitis, and abdominal tuberculous lymphadenitis [105]. Eighty percent of them are from reactivation of the LTBI and twenty percent of them come from new infections in contact with tuberculosis patients [100]. Compared with immunosuppression caused by other factors, IGRAs seem to be less affected by ESRD but have no value as a single test either to rule in or rule out ATBI [106].

#### 2.2.5. End-Stage Liver Disease

LTBI in patients with end-stage liver disease (ESLD) is higher than the general population and has a 29.5 times higher risk of developing into ATBI after liver transplantation [81]. The performers of the TST and IGRAs are impaired because of patients’ T-cell anergy. Most studies showed that the concordance between the two was high, but the positive rate of IGRAs was higher in worse liver disease. A study from 153 patients with ESLD waiting for liver transplantation found that the concordance between IGRAs and the TST was 85.1% [107]. TST+/IGRA− results are mostly due to the influence of BCG. However, it should be noted that patients with a positive TST from remote vaccination are more likely to develop an infection than cross-reaction with BCG [85]. IGRAs perform better in worse liver diseases, even compared with a two-step TST (boosted TST). One study showed that the positive rate of the TST decreased significantly in patients with a model for end-stage liver disease (MELD) score of more than 18 or Child–Pugh–Turcotte grade C, and the positive rate of IGRAs did not decrease significantly [81]. However, worse liver disease leads to an increase of indeterminate IGRAs results (2–40.6%), especially when the MELD score was higher than 25 [108]. For every 1-point increase in MELD score, the indeterminate result increased by 7% [109]. A simultaneous TST and IGRA rather than a two-step IGRA or a two-step TST could be a better choice [81].

#### 2.2.6. Diabetes Mellitus

Diabetes mellitus (DM) is undoubtedly a risk factor, with a 15% higher risk of LTBI [110] and a 3 times increased risk of ATBI [111]. It is also a risk of death, delayed sputum conversion, treatment failure, relapse, and rifampicin resistance [111]. In some regions of the world, up to 22% of tuberculosis cases can be attributed to diabetes [112]. Factors that contribute to tuberculosis infection include the following: (1) blood glucose dysregulation leads to hyperglycemia and insulin resistance, which enhances the replication of *M. tuberculosis*, and (2) the presence of free fatty acids and lipid intermediates in monocytes promotes an inflammatory environment to progress to tuberculosis [111].

Interestingly, ATBI in DM has unique characteristics. For example, cavitary tuberculosis is common in diabetic patients but rare in HIV-infected patients [111]. Unlike other immunocompromised, patients with diabetes show their capacity to secrete high levels of IFN-γ. Therefore, diabetes does not affect the sensitivity of IGRAs [113] and the proportion of indeterminate results in diagnosing LTBI [114].

### 2.3. Worldwide Guidelines

Since 2003, 25 countries and two supranational organizations (WHO and European Centre for Disease Prevention and Control) have published the clinical application guidelines or position statements of IGRAs for diagnosing tuberculosis infection. There are some differences between the recommendations from various countries, due to their different local situations (tuberculosis prevalence, economic status, immigration number, BCG coverage, etc.). Table 1 summarizes several main international or national guidelines on the use of IGRAs and the TST in the immunocompromised population [48,49,93,96,115].

### 2.4. Prospects

To address the imperfections of traditional IGRAs in the immunocompromised population, there are several novel tests under investigation that promise to improve performers. New generations of IGRAs have developed that can differentiate T-cell subsets. A different subset of T-cells secretes different cytokines: central memory T-cells secrete interleukin (IL)-2, effector memory T-cells secrete IFN-γ and IL-2, and effector T-cells secrete IFN-γ. Whereas IL-2 is associated with a low antigen load and LTBI, IFN-γ is associated with a high antigen load and conversion from LTBI to ATBI. Therefore, the new IGRAs could better predict the risk of tuberculosis progression in this population [116,117]. Further, some studies used other biomarkers, with IP-10 release assays being the most investigated [117]. Studies showed that IP-10 appeared to be less affected by low CD4 + T-cells than IFN-γ. Among patients with HIV, it was reported that IP-10 was more sensitive than IGRAs in ATBI and LTBI [118,119].

## 3. Conclusions

In conclusion, the immunocompromised situation caused by both physiological and pathological factors affects the accuracy of IGRAs in varying degrees, including reducing sensitivity and specificity and increasing intermediate results. In combination with other detection technology, IGRAs have some diagnostic value for LTBI in the immunocompromised population and ATBI in children. The newly developed IGRA products are expected to better target this population.

## Figures and Tables

**Table 1:** Worldwide guidelines on recommendations of interferon-gamma release assays (IGRAs) and the tuberculin skin test (TST) for tuberculosis infection in the immunocompromised population.

	WHO (International) ^1^	NTAC/CDC (The United State) ^2^	NICE (Britain) ^3^	SEIMC/SEPAR (Spain) ^4^
Latent tuberculosis infection (LTBI)				
Pregnant and puerperal women	Not mentioned	Not mentioned	Not mentioned	Not mentioned
Older people	Not mentioned	Not mentioned	Not mentioned	Not mentioned
Children				
>5 years old	Either (contacts of patients with ATBI)	Either, but IGRAs preferred	IGRAs when the initial TST is negative (contacts of patients with ATBI)	TST (contacts of patients with ATBI)
<5 years old	Either (contacts of patients with ATBI)	Either, but TST preferred	IGRAs when the initial TST is negative (contacts of patients with ATBI)	Both (contacts of patients with ATBI)
Human immunodeficiency virus infection				
CD4 + T-cell < 200 cells/μL	Either	Either	IGRAs or IGRAs + TST	Both
CD4 + T-cell < 200 cells/μL	Either	Both	IGRAs + TST	IGRAs
Solid organ transplant recipients	Either	Either (low-risk) Both (high-risk)	IGRAs + TST	Both
Chronic immuno-mediated inflammatory diseases	Either (patients receiving tumor necrosis factor-α blockers)	Either (low-risk) Both (high-risk)	IGRAs or IGRAs + TST (patients receiving tumor necrosis factor-α blockers)	Both
End-stage renal disease	Either (patients receiving dialysis)	Either (low-risk) Both (high-risk)	IGRAs or IGRAs + TST	Not mentioned
End-stage liver disease	Not mentioned	Either (low-risk) Both (high-risk)	Not mentioned	Not mentioned
Diabetes mellitus	No screening is required	Not mentioned	IGRAs or IGRAs + TST	Not mentioned
Active tuberculosis infection (ATBI)	Neither	Both as adjunctive tests for children	Both as complementary tests for children <15 years old	Both as complementary tests for children >5-year-old

^1^ World Health Organization. Its recommendations are limited to the high/middle-income countries. In the low/middle-income countries, IGRAs should not replace the TST. ^2^ National Tuberculosis Advisory Committee/Centers for Disease Control and Prevention. ^3^ National Institute for Health and Care Excellence. ^4^ Spanish Society of Infectious Diseases and Clinical Microbiology/Spanish Society of Respiratory Diseases and Thoracic Surgery.

## Data Availability

Not applicable.
